# Optimization methods for the distortion of thin-walled box girders and investigation of distortion effects

**DOI:** 10.1038/s41598-023-46478-1

**Published:** 2023-11-06

**Authors:** Zhaonan Wang

**Affiliations:** Department of Civil Engineering, Lanzhou City University, Lanzhou, 730070 Gansu China

**Keywords:** Engineering, Civil engineering

## Abstract

To investigate the distortion process of thin-walled box girders, three commonly used methods based on energy variational calculus and static balance analysis are optimized. A generalized analytical formula for box-girder distortion research is derived, and a fourth-order distortion control differential equation is obtained. Typical numerical examples are used to verify and compare the three methods. The results show that the value of distortional warping normal stress calculated by the optimized methods is slightly different from the literature values and that the maximum error between methods does not exceed 5.39%. The calculation results of Method 2 and Method 3 are similar. The values of the geometric distortion characteristics calculated by the three methods are related to the cross-sectional form of the box girder and the distortion analysis process, and the calculated values are not unique. The absolute value of the peak normal stress of distortion on the top plate of a thin-walled box girder with a cantilever plate is smaller than that on the bottom plate. Under a concentrated distortion load, the distribution of the distortion deformation along the length of a simply supported box girder with only end diaphragm is not consistent, and there is reverse deformation near the beam end.

## Introduction

Distortion effects play a significant role in the spatial mechanics of thin-walled box girders. Under eccentric vertical loads, the distortion warping normal stress of a box girder far exceeds the torsion warping normal stress in proportion to the total longitudinal normal stress. Based on this, many studies have been conducted on the distortion of thin-walled box girders. The research methods can basically be divided into energy variational calculus based on the principle of stationary potential energy and plate element analysis methods based on the static balance analysis of each plate in a box girder.

Guo et al.^1^ introduced the application of plate element analysis methods to study the distortion of box girders with a rectangular cross section. Energy variational calculus has the advantage of clear physical concepts, but the analysis process is relatively complex^2,3^. Kermani et al.^4^ analyzed the distortion effect of a two-span continuous box girder with equal cross section using the energy variation calculus. Boswell et al.^5^ used energy variational calculus to study the torsion and distortion of curved box girders. The distribution of distortion sector coordinates on the cross section of curved box girders follows a curve in the form of. In literatures^6–9^ used the energy variation calculus to study the deformation of box girders. Zhang et al.^7^ used energy variational calculus to study the distortion effect of box girders and analyzed the distribution of the distortion moment, distortion warpage rate, and other factors as a function of the box-girder length. Regardless of the distortion analysis method used, the calculation results for the distortion warping normal stress are similar. Marcello et al.^10^ analysed the distortion effect of curved box girders using the Hamiltonian Structural Analysis method. Generally, shell element, solid element and 3-D beam element are used to analyze the distortion effect of box girder^11,12^, which is more applicable, but the strict logic and the convenience of parameter research are not as good as the theoretical analysis method. In literatures^13,14^, the distortion of single box double-cell box girders and the effect of transverse diaphragms on box girder deformation were examined. Li et al.^15^ studied the distortion effect of a variable-height box girder bridge by using the plate element analysis method and solved the distortion differential equation via the Newmark method. Usually, the solution of distortion differential equations is achieved by using analogous beam on elastic foundation solutions and initial parameter solutions^16–20^.

The process of energy variation calculus based on the principle of stationary potential energy is complex, but the physical meaning of the relevant variables is relatively clear. The process of plate element analysis based on static equilibrium analysis is relatively simple. In this study, three distortion analysis methods based on these two principles are optimized and investigated. Multiple numerical examples are used to compare the differences in the distortion characteristics between each method and between straight and inclined web box girders. The differences in application between two commonly used methods for solving distortion differential equations, namely, the analogous beam on elastic foundation solutions and the initial parameter solutions, are clarified. Finally, a detailed study was conducted regarding the distribution of distortion angles, distortion moments, and other factors along the length of the box girder.

## Distortion load and general section form of box girders

**Figure 1:**
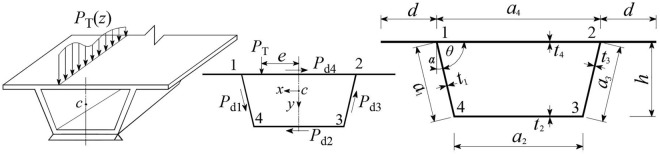
Eccentric vertical load and cross section of the box girder.

The eccentric vertical load *P*_T_(*z*) acting on the top plate of the thin-walled box girder is shown in Fig. 1. The vertical load *P*_T_ acts along the *y*-axis, and the horizontal distance from the centroid *c* of the section is *e*. The *P*_T_ can be decomposed into moments acting on the top plate, causing torsion and distortion of the box girders. The moment can be decomposed into an antisymmetric load *P* = *P*_T_*e*/*a*_4_ acting on the connection point between the web and top plate. Then, the antisymmetric load *P* can be decomposed to obtain the distortion load on the left and right web plates, namely, $P_{{{\text{d1}}}} = P_{{{\text{d3}}}} = Pa_{{2}} a_{{1}} /\left[ {\left( {a_{{4}} + a_{{2}} } \right)h} \right]$. The distortion load on the bottom plate is $P_{{{\text{d2}}}} = Pa_{{4}} a_{{2}} /\left[ {\left( {a_{{4}} + a_{{2}} } \right)h} \right]$, and the distortion load on the top plate is $P_{{{\text{d4}}}} = Pa_{2}^{2} /\left[ {\left( {a_{{4}} + a_{{2}} } \right)h} \right]$, as shown in Fig. 1.

In Fig. 1, the corners of the box girder are represented by numbers 1 ~ 4, *h* represents the beam height, *a*_*i*_ and *t*_*i*_ (*i* = 1, 2, 3, 4) correspond to the width and thickness of the left web, bottom plate, right web, and top plate, respectively, *a*_1_ = *a*_3_, *t*_1_ = *t*_3_, and *d* is the width of the cantilever plate.* I*_1_ = *I*_3_, *I*_2_ and *I*_4_ are defined as the out-of-plane inertia moments of the web, bottom, and top plates of the box girder, respectively. $I_{i} = t_{i}^{3} /\left[ {{12}\left( {{1} - \mu^{{2}} } \right)} \right] \, \left( {i = {1},{2},{4}} \right)$, where *μ* is Poisson's ratio. *J*_1_ = *J*_3_, *J*_2_, and *J*_4_ are the in-plane inertia moments of each plate element. $J_{i} = t_{i} a_{i}^{\rm{3}} /{12}$ (*i* = 1, 2), and $J_{{4}} = t_{{4}} \left( {a_{{4}} + {2}d} \right)^{{3}} /{12}$. *α* is the dip angle of the web plate, and *θ* is the angle between the web and top plate, with the coordinate origin located at *c*, the right-hand coordinate system. The distortion assumption of box girders can be found in the literature^2,7^.

## Distortion research method based on distortion sector coordinate analysis

### Distortion sector coordinate and box girder warping strain energy

**Figure 2:**
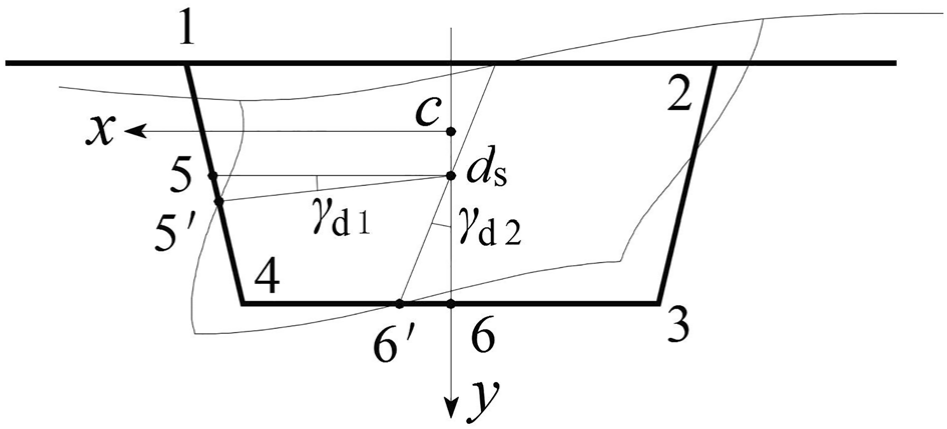
Distortional angle and deformation of the box girder.

Under an eccentric vertical load, the box girder section deforms around the centre of distortion *d*_s_. The tangential displacement *v*(*s*) is defined as positive in the counterclockwise direction around the distortion centre *d*_s_ of the box girder cross section. As shown in Eq. (1), each item represents the tangential displacement of the top, bottom, and web plate. Here, *y*_c_ is the distance from the centreline of the top plate to the centroid *c* of the box girder section, *y*_d_ is the distance from the distortion centre *d*_s_ of the box girder to the centroid *c* of the cross section, and *x*_1_ is the *x*-coordinate of 5 points on the web plate. After the distortion of the box girder, the displacement from point 5 to point 5′ and from point 6 to point 6′, the angle formed by the connection between these two points and *d*_s_ is *γ*_d1_, *γ*_d2_. The distortion angle of this method is defined as $\gamma_{{\text{d}}} = \gamma_{{{\text{d1}}}} + \gamma_{{{\text{d2}}}}$, as shown in Fig. 2.

1 $$v(s) = \left\{ \begin{gathered} - (y_{\rm{c}} + y_{\rm{d}} )\gamma_{{\rm{d2}}} \hfill \\ - (h - y_{\rm{c}} - y_{\rm{d}} )\gamma_{{\rm{d2}}} \hfill \\ x_{1} \gamma_{{\rm{d1}}} \cos \alpha \hfill \\ \end{gathered} \right.$$

According to the assumption, the tangential mid-plane shear strain of the box girder *γ*_zs_ = 0, with $\oint {\frac{\partial v}{{\partial z}}} = 0$. After substituting Eq. (1), the following can be obtained:

2 $$\gamma_{\rm{d}} = \frac{{(1 + k_{\rm{d}} )\gamma_{{\rm{d2}}} }}{{k_{\rm{d}} }} = (1 + k_{\rm{d}} )\gamma_{{\rm{d1}}}$$

where $k_{{\text{d}}} = \frac{{2x_{1} h}}{{(y_{{\text{c}}} + y_{{\text{d}}} )a_{4} + (h - y_{{\text{c}}} - y_{{\text{d}}} )a_{2} }}$. Due to $\frac{\partial v}{{\partial z}} + \frac{\partial w}{{\partial s}} = 0$,* w*(*s*) is the *z*-axis distortion warping displacement. By integrating along the tangential direction of the box girder section, it can be obtained that $w(s) = \int_{0}^{s} {\left( { - \frac{\partial v}{{\partial z}}} \right)} {\text{d}}s + C^{\prime }$. Proper selection of the calculation point can make the constant of integration $C^{\prime }$ as 0, and Eq. (1) is substituted into *w*(*s*) to obtain:

3 $$w(s) = \left\{ \begin{gathered} \int_{0}^{s} {\frac{{(y_{\rm{c}} + y_{\rm{d}} )k_{\rm{d}} }}{{1 + k_{\rm{d}} }}} \gamma_{{\text{d}}}^{\prime } \rm{d}s \hfill \\ \int_{0}^{s} {\frac{{(h - y_{\rm{c}} - y_{\rm{d}} )k_{\rm{d}} }}{{1 + k_{\rm{d}} }}} \gamma_{{\text{d}}}^{\prime } \rm{d}s \hfill \\ - \int_{0}^{s} {\frac{{x_{1} \cos \alpha }}{{1 + k_{\rm{d}} }}} \gamma_{{\text{d}}}^{\prime } \rm{d}s \hfill \\ \end{gathered} \right.$$

Equation (3) represents the *z*-axis distortion warping displacement of the top, bottom, and web plate. If Eq. (3) is represented as $w(s) = \int_{0}^{s} {\rho_{{{\text{dd}}}} {\text{d}}s \cdot \gamma_{{\text{d}}}^{\prime } = \omega_{{\text{d}}} \cdot \gamma_{{\text{d}}}^{\prime } }$, then $\omega_{{\text{d}}} = - \int_{0}^{s} {\rho_{{{\text{dd}}}} {\text{d}}s}$, and *ω*_d_ can be defined as the distortion sector coordinate of a box girder. The distortion normal stress of the box girder can be obtained by the following:

4 $$\sigma_{{\text{d}}} (s) = E\frac{\partial w}{{\partial s}} = E\omega_{{\text{d}}} \gamma_{{\text{d}}}^{\prime \prime }$$

In Eq. (4), *E* is the material elastic modulus. Definition *ω*_d1_ and *ω*_d4_ are the distortion sector coordinates of points 1 and 4 of the box girder, respectively. *σ*_d1_ and *σ*_d4_ are the distortion normal stress values at points 1 and 4 of the box girder, respectively. Let *β* = *σ*_d1_/*σ*_d4_, according to the stress self-equilibrium condition, it can be concluded that *β* value. From the definition of *ω*_d_, $\omega_{{\rm{d}1}} { = }\frac{{ - k_{\rm{d}} (y_{\rm{c}} + y_{\rm{d}} )a_{4} }}{{2(1 + k_{\rm{d}} )}}$, and $\omega_{{{\text{d}}4}} { = }\frac{{k_{\rm{d}} (h - y_{\rm{c}} - y_{\rm{d}} )a_{2} }}{{2(1 + k_{\rm{d}} )}}$.

$I_{{\omega d}} { = }\int_{F} {\omega_{\rm{d}}^{2} } {\text{d}}F$ is defined as the distortion warping sector moment of inertia of the box girder in the distortion normal stress self-balance analysis, and *F* is the cross-sectional area of the box girder. Then,

5 $$I_{{\omega d}} { = }\frac{{(a_{4} + 2d)^{3} t_{4} + 2a_{4}^{2} a_{1} t_{1} }}{{6a_{4}^{2} }}\omega_{{\rm{d}1}}^{2} + \frac{{(a_{2} t_{2} + 2a_{1} t_{1} )}}{6}\omega_{{\rm{d4}}}^{2} - \frac{{a_{1} t_{1} }}{3}\omega_{{\rm{d}1}} \omega_{{\rm{d}4}}$$

Therefore, the distortion warping strain energy of the box girder is $\frac{1}{2}\int_{L} {EI_{{\omega d}} (\gamma_{{\text{d}}}^{\prime \prime } } )^{2} \rm{d}z$, where the integral path along the *z*-axis of the box girder is the girder length *L.*

### Box girder transverse bending strain energy

When the box girder produces a distortion, the transverse bending moments at corner points 1 and 4 of the box girder are *M*_1_ and *M*_4_. Through calculation,* M*_1_ and *M*_4_ can be expressed as a linear relationship between the distortion transverse bending moment of the plate and the distortion angle *γ*_d_ of the box girder. Here, $M_{\rm{1}} = EI_{{\rm{R}1}} \gamma_{\rm{d}}$, $M_{\rm{4}} = EI_{{\rm{R}3}} \gamma_{\rm{d}}$, and *I*_R1_ and *I*_R3_ are coefficients that generate the transverse bending moment of inertia *I*_R_ of the box girder. The transverse bending strain energy of the box girder can be obtained as follows:

6 $$U_{1} = \int_{L} {\int_{s} {\frac{{M^{2} (s)}}{2EI}} } {\text{d}}s\rm{d}z = \frac{E}{2}\int_{L} {I_{\rm{R}} \gamma_{\rm{d}}^{2} \rm{d}z}$$

where $I_{\rm{R}} = \frac{{a_{4} I_{{\rm{R}1}}^{2} }}{{3I_{4} }} + \frac{{a_{2} I_{{\rm{R}3}}^{2} }}{{3I_{2} }} + \frac{{2a_{1} (I_{{\rm{R}1}}^{2} + I_{{\rm{R}3}}^{2} + I_{{\rm{R}1}} I_{{\rm{R}3}} )}}{{3I_{1} }}$ in m^2^, *M*(*s*) is the transverse bending moment of the frame, and *s* is the circumferential coordinate of the closed part of the box girder section.

### Distortion control differential equation of the box girder

The sum of the distortion warping strain energy and transverse bending strain energy of the box girder is shown in Eq. (7) as follows:

7 $$U_{{\rm{12}}} = \frac{1}{2}\int_{L} {[EI_{{\omega d}} (\gamma^{\prime\prime}_{\rm{d}} )^{2} + EI_{\rm{R}} \gamma_{\rm{d}}^{2} ]} \rm{d}z$$

The distortion external load potential energy of the box girder is shown in Eq. (8):

8 $$U_{3} = - \int_{L} {m_{\rm{d}} \gamma_{\rm{d}} } \rm{d}z$$

where $m_{{\text{d}}} = 2P\frac{{x_{1} + k_{{\text{d}}} ( - y_{{\text{c}}} - y_{{\text{d}}} )\tan \alpha }}{{1 + k_{{\text{d}}} }}$. Then, the total distortion potential energy of the box girder can be obtained by the following:

9 $$U = \frac{1}{2}\int_{L} {\left[ {EI_{{\omega d}} (\gamma_{{\text{d}}}^{\prime \prime } )^{2} + EI_{\rm{R}} \gamma_{\rm{d}}^{2} - 2m_{\rm{d}} \gamma_{\rm{d}} } \right]} \rm{d}z$$

The unit length box girder in the middle of the span is taken along the longitudinal direction of the box girder. According to the variational principle, the Euler equation is used to yield $\lambda = \left[ {I_{{\text{R}}} /\left( {{4}I_{{\omega {\text{d}}}} } \right)} \right]^{{{1}/{4}}}$, and the distortion control differential equation can be obtained as follows:

10 $$\gamma_{{\text{d}}}^{\prime \prime \prime \prime } + 4\lambda^{4} \gamma_{\rm{d}} = m_{\rm{d}} \rm{/(}EI_{{\omega d}} \rm{)}$$

The differential equation can be solved by the initial parameter solutions. The odd form of Eq. (10) is $\gamma_{{\text{d}}}^{\prime \prime \prime \prime } + 4\lambda^{4} \gamma_{\rm{d}} = 0$. Define $B_{\rm{d}} = EI_{{\omega d}} \gamma_{{\text{d}}}^{\prime \prime }$, then, the distortion normal stress can be expressed as $\sigma_{\rm{d}} = B_{\rm{d}} \omega_{\rm{d}} /I_{{\omega d}}$, where *B*_d_ is the distortion warping bimoment of the box girder. This is distortion analysis Method 1 of the box girder.

## Distortion research method based on the energy variational calculus

Single box single-cell box girders generally have a symmetry axis, so the distortion transverse bending moment at each corner of the box girder is conveniently expressed by the distortion angle, and then the expression of the transverse bending moment of inertia of the box girder can be obtained. Via this method, the transverse bending moment of inertia is obtained by solving the transverse bending moment of the box girder. The obtained distortion warping sector moment of inertia of the box girder is also different from that obtained by setting the circumferential displacement. The distortion warping sector moment of inertia is derived by using the relationship between the displacement of each corner after the distortion of the box girder and the in-plane moment.

### Transverse bending strain energy of the box girder

**Figure 3:**
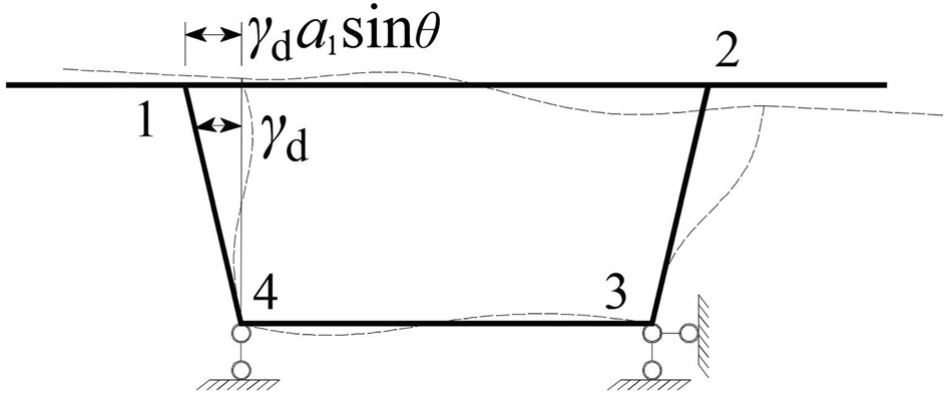
Deformation of the box girder after horizontal lateral displacement.

The distortion angle of the box girder in this method is defined as *γ*_d_, as shown in Fig. 3. When the box girder is distorted, the distortion angle is *γ*_d_, and the horizontal displacement of the top slab is *γ*_d_*h*, which can also be expressed as *γ*_d_*a*_1_sin*θ*.

When the unit horizontal force $\overline{P} = 1$ acts at the midspan of the top slab of the box girder, the shear force *X*_1_ at the midspan of the top slab and the horizontal lateral displacement of the top slab *δ*_h_ can be obtained through the force method equation, as shown in Eqs. (11) and (12).

11 $$X_{1} { = }\frac{{2[a_{2}^{2} hI_{1} I_{4} + a_{1} h(a_{4} + 2a_{2} )I_{2} I_{4} ]}}{{a_{4}^{3} I_{1} I_{2} + a_{2}^{3} I_{1} I_{4} + 2a_{1} (a_{4}^{2} + a_{2}^{2} + a_{2} a_{4} )I_{2} I_{4} }} = 2X$$

12 $$\delta_{\rm{h}} { = }\left[ {\frac{{a_{4}^{3} }}{{I_{4} }} + \frac{{a_{2}^{3} }}{{I_{2} }} + \frac{{2a_{1} (a_{4}^{2} + a_{2}^{2} + a_{2} a_{4} )}}{{I_{1} }}} \right]\frac{{X^{2} }}{6E} - \left[ {\frac{{a_{2}^{2} }}{{I_{2} }} + \frac{{a_{1} (a_{4} + 2a_{2} )}}{{I_{1} }}} \right]\frac{hX}{{3E}} + \left( {\frac{{a_{2} }}{{I_{2} }} + \frac{{2a_{1} }}{{I_{1} }}} \right)\frac{{h^{2} }}{6E}$$

Then, the vertical shear force in the midspan of the top slab of the box girder shown in Fig. 3 is $\gamma_{\rm{d}} a_{1} X_{1} \sin \theta /\delta_{{\text{h}}}$, the transverse bending moment at point 1 of the frame is $M_{1} = \gamma_{\rm{d}} a_{1} a_{4} X\sin \theta /\delta_{{\text{h}}}$, and the transverse bending moment at point 4 of the frame is $M_{4} = \gamma_{\rm{d}} a_{1} (a_{2} X - a_{1} \sin \theta )\sin \theta /\delta_{{\text{h}}}$. The distortion angle *γ*_d_ is used to represent the distortion transverse bending moment at the corner of the box girder, so the transverse bending strain energy of the unit length box girder segment can be expressed as:

13 $$U_{1} = \frac{{M_{1}^{2} a_{4} }}{{6EI_{4} }} + \frac{{M_{4}^{2} a_{2} }}{{6EI_{2} }} + \frac{{2a_{1} (M_{1}^{2} + M_{4}^{2} + M_{1} M_{4} )}}{{6EI_{1} }}$$

Substituting the expressions of *M*_1_ and *M*_4_ into Eq. (13) yields

14 $$U_{1} { = }EI_{\rm{R}} \gamma_{\rm{d}}^{2}$$

where $I_{\rm{R}} = 6\frac{{\frac{{a_{4}^{3} }}{{I_{4} }} + \frac{{a_{2}^{3} }}{{I_{2} }} + \frac{{2a_{1} (a_{4}^{2} + a_{2}^{2} + a_{2} a_{4} )}}{{I_{1} }}}}{{\frac{{a_{2} a_{4}^{3} }}{{I_{2} I_{4} }} + \frac{{2a_{1} a_{4}^{3} }}{{I_{1} I_{4} }} + \frac{{2a_{1} a_{2} a_{4}^{2} }}{{I_{1} I_{2} }} + \frac{{3a_{1}^{2} a_{4}^{2} }}{{I_{1}^{2} }}}}$, unit is m^2^.

### Distortion warping strain energy of the box girder

**Figure 4:**
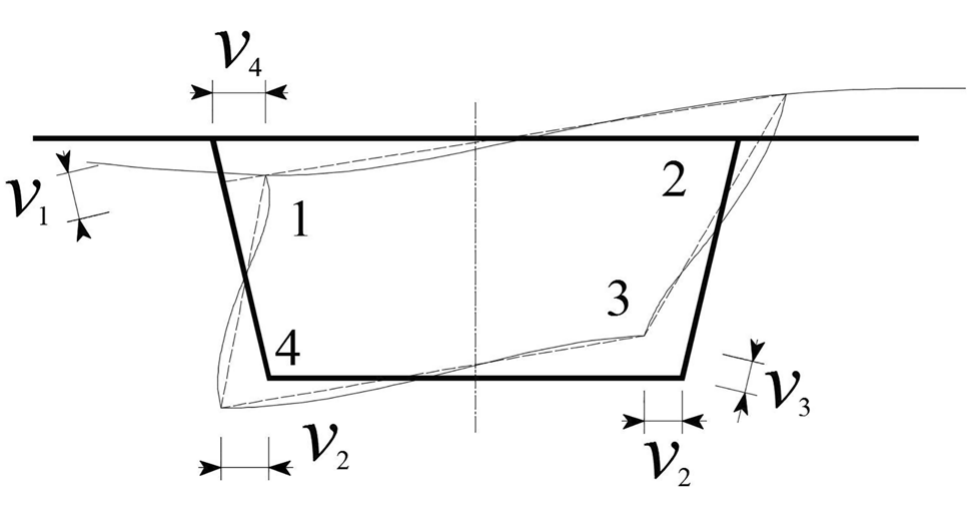
Distortional deformation and corner displacement of the box girder.

The displacement *v*_*i*_ of each corner point after the distortion of the box girder in this method is defined as shown in Fig. 4. According to the relationship between the deflection of the elementary beam and the bending moment, *v*_*i*_ (*i* = 1, 2, 3, 4) can be obtained. Therefore, the quadratic differential of each corner displacement can be expressed as $v^{\prime\prime}_{1} = v^{\prime\prime}_{3} = - \frac{1 + \beta }{{Ea_{1} }}\sigma_{{\rm{d}4}}$, $v^{\prime\prime}_{2} = - \frac{{2\sigma_{{\rm{d4}}} }}{{Ea_{2} }}$, $v^{\prime\prime}_{4} = - \frac{{2\beta \sigma_{{\rm{d}4}} }}{{Ea_{4} }}$.

The distortion angle *γ*_d_ of the box girder in Fig. 3 can be represented by the displacement of each corner of the box girder^2^ in Fig. 4:

15 $$\gamma_{\rm{d}} = \frac{{\rm{d}x_{1} - \rm{d}x_{2} }}{{a_{1} \sin \theta }} + \frac{{\rm{d}y_{3} - \rm{d}y_{2} }}{{a_{2} }}$$

Through the analysis of Fig. 4, can obtain the following results, namely, ${\text{d}}x_{1} = v_{4} ,\;{\text{d}}x_{2} = - \;v_{2} ,\;{\text{d}}y_{3} = \frac{{v_{2} }}{\tan \theta } + \frac{{v_{3} }}{\sin \theta },$ and $- {\text{d}}y_{2} = \frac{{v_{1} }}{\sin \theta } + \frac{{v_{2} }}{\tan \theta }$, which is simplified by substituting it into Eq. (15). Additionally, the distortion angle *γ*_d_ is differentiated twice about the *z*-axis, and then, $v_{i}^{{\rm{\prime \prime }}}$ (*i* = 1,2,3,4) is substituted, which is obtained by combining Eq. (4).

16 $$\sigma_{{\rm{d}4}} { = } - E\omega_{{\rm{d}4}} \gamma_{{\text{d}}}^{\prime \prime }$$

where $\omega_{{\rm{d}4}} = \frac{{a_{2}^{2} a_{4} h}}{{2a_{2} a_{4} (1 + \beta ) + 2a_{2}^{2} \beta + 2a_{4}^{2} }}$ and $\sigma_{{\rm{d1}}} = - E\omega_{{\rm{d}4}} \beta \gamma_{{\text{d}}}^{\prime \prime }$. Here, the distortion warping normal stress is substituted into the strain energy formula with respect to the normal stress *σ*_d1_. Taking the unit length of the box girder, the distortion warping strain energy of the box girder can be obtained as:

17 $$U_{2} = EI_{{\omega d}} (\gamma_{{\text{d}}}^{\prime \prime } )^{2}$$

where $I_{{\omega d}} { = }\frac{{\left[ {(a_{4} + 2d)^{3} a_{2}^{4} t_{4} \beta^{2} + a_{2}^{5} a_{4}^{2} t_{2} } \right]h^{2} + 2a_{1} a_{2}^{4} a_{4}^{2} h^{2} t_{1} (1 + \beta^{2} - \beta )}}{{24[a_{2} a_{4} (1 + \beta ) + a_{2}^{2} \beta + a_{4}^{2} ]^{2} }}$, unit is m^6^.

### Distortion load potential energy and distortion control differential equation

In this method, the potential energy of the box girder under a distorted load can be expressed as $U_{3} = - \int_{0}^{L} {P_{{\rm{d}4}} h\gamma_{\rm{d}} \rm{d}z}$. Taking the unit length of the box girder, the total potential energy of the frame when the box girder is distorted can be represented as:

18 $$U = EI_{{\omega d}} (\gamma_{{\text{d}}}^{\prime \prime } )^{2} + EI_{\rm{R}} \gamma_{\rm{d}}^{2} - \frac{{Pa_{2}^{2} }}{{(a_{2} + a_{4} )}}\gamma_{\rm{d}}$$

Based on the energy variational principle and Euler equation, the distortion governing differential equation of the box girder is obtained as:

19 $$EI_{{\omega d}} \gamma_{{\text{d}}}^{\prime \prime \prime \prime } + EI_{\rm{R}} \gamma_{\rm{d}} = Pa_{2}^{2} /[2(a_{2} + a_{4} )]$$

The distortion normal stress *σ*_d_ can be calculated after the corresponding quantity is obtained, and the distortion shear stress *τ*_d_ can be calculated according to the equilibrium condition of the element. In this section, $\gamma_{{\text{d}}}^{\prime \prime } = - B_{\rm{d}} /(EI_{{\omega d}} )$, and the expression of the distortion normal stress of corner 4 can be obtained as $\sigma_{{\rm{d4}}} = B_{\rm{d}} \omega_{{\rm{d}4}} /I_{{\omega d}}$. This is the box-girder distortion analysis Method 2.

## Distortion research method based on the static equilibrium of plate elements

Different from the former two methods, this method is based on the static equilibrium analysis of the plates of the box girder under an eccentric vertical load, and the plate element analysis method is used to obtain the distortion differential equation of the box girder. The form of the box girder is shown in Fig. 1. This method defines the sum of the changes in the angle between the web and bottom plate connected to corner 4 of the box girder as the distortion angle *γ*_d_ of the box girder.

### Analysis of the in-plane force system of each plate of the box girder

**Figure 5:**
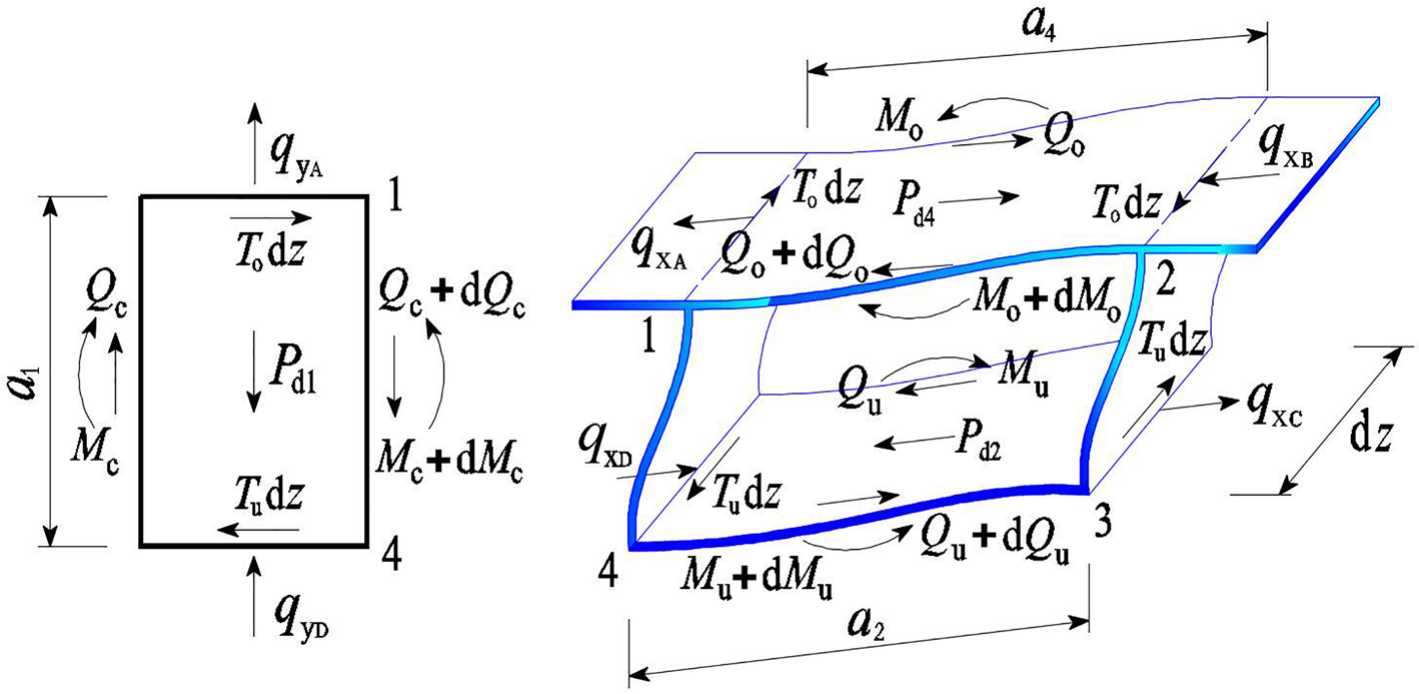
Force system of the top and bottom slab and web.

After distortion of the box girder, the force in the analysis plane is shown in Fig. 5. Through the in-plane static balance analysis of each plate of the box girder, ignoring the small amount in the balance equation, the in-plane force system balance equation of each plate can be obtained, as shown in Eq. (20).

In Fig. 5, *q*_xB_ and *q*_xA_ are the transverse binding forces of the box girder web to the top plate, *T*_o_d*z* is the longitudinal binding force of the web to the top plate, *P*_d4_ is the in-plane distortion load of the top plate, and d*Q*_o_ and d*M*_o_ are the in-plane shear force and moment increment generated on the micro segment of the top plate, respectively. *q*_xC_ and *q*_xD_ are the transverse binding forces of the box girder web to the bottom plate, *T*_u_d*z* is the longitudinal binding force of the web to the bottom plate, *P*_d2_ is the in-plane distortion load of the bottom plate, and d*Q*_u_ and d*M*_u_ are the in-plane shear force and moment increment generated on the micro segment of the bottom plate, respectively. *q*_yA_ and *q*_yD_ are the transverse binding forces of the top plate and bottom plate of the box girder to the left web, *T*_o_d*z* and *T*_u_d*z* are the longitudinal binding forces of the top and bottom plate on the micro segment to the web, d*Q*_c_ and d*M*_c_ are the in-plane shear force and moment increment generated on the micro segment of the left web, respectively, and *P*_d1_ is the in-plane distortion load of the left web.

20 $$\frac{{\rm{d}^{2} M_{\rm{c}} }}{{\rm{d}z^{2} }} + \frac{{a_{1} }}{{2a_{4} }}\frac{{\rm{d}^{2} M_{\rm{o}} }}{{\rm{d}z^{2} }} + \frac{{a_{1} }}{{2a_{2} }}\frac{{\rm{d}^{2} M_{\rm{u}} }}{{\rm{d}z^{2} }} + \frac{{a_{1} }}{{2a_{4} }}P_{{\rm{d4}}} + \frac{{a_{1} }}{{2a_{2} }}P_{{\rm{d}2}} + P_{{\rm{d1}}} - \left( {q_{\rm{y}} + \frac{{a_{1} }}{{2a_{4} }}q_{{\rm{x}1}} + \frac{{a_{1} }}{{2a_{2} }}q_{{\rm{x}2}} } \right) = 0$$

where $q_{{\text{y}}} = q_{{{\text{yA}}}} + q_{{{\text{yD}}}} ,\;q_{{{\text{x2}}}} = q_{{{\text{xC}}}} + q_{{{\text{xD}}}} ,\;{\text{and}}\;q_{{{\text{x1}}}} = q_{{{\text{xB}}}} + q_{{{\text{xA}}}}$. From the relationship between the distortion warping normal stress at each corner of the box girder and the in-plane moment of each plate, the relationship between the in-plane moment of the top, bottom and web plate can be obtained as follows: $M_{{\text{o}}} = \frac{{2a_{1} J_{4} M_{\rm{c}} }}{{\left( {1 + \beta } \right)a_{4} J_{1} }}$, $M_{\rm{u}} = \frac{{2\beta a_{1} J_{2} M_{\rm{c}} }}{{\left( {1 + \beta } \right)a_{2} J_{1} }}$. From the relationship between the bending moment and deflection via elementary beam theory, *γ*_d_ and *M*_c_ have the following relationship:

21 $$\frac{{\rm{d}^{2} M_{\rm{c}} }}{{\rm{d}z^{2} }} = \frac{{\gamma _{\rm{d}}^{{\prime \prime \prime \prime }} }}{{\chi _{1} }}$$

where $\chi_{1} = \frac{{ - 2a_{1} (a_{2} + a_{4} )(a_{2} + a_{4} \beta )}}{{EJ_{1} \left( {1 + \beta } \right)a_{4} a_{2}^{2} h}}$. *M*_o_, *M*_u_ and Eq. (21) are substituted into Eq. (20) to obtain the description for distortion angle *γ*_d_ and the differential equation of the force system on each plate, as shown in Eq. (22).

22 $$- \frac{{EI_{{\rm{\omega d}}} }}{{a_{4} }}\gamma _{\rm{d}}^{{\prime \prime \prime \prime }} + \frac{{a_{1} P_{{\rm{d4}}} }}{{2a_{4} }} + \frac{{a_{1} P_{{\rm{d}2}} }}{{2a_{2} }} + P_{{\rm{d1}}} - \left( {q_{\rm{y}} + \frac{{a_{1} q_{{\rm{x}1}} }}{{2a_{4} }} + \frac{{a_{1} q_{{\rm{x}2}} }}{{2a_{2} }}} \right) = 0$$

where $I_{{\omega d}} = \frac{{h\left[ {\left( {1 + \beta } \right)a_{2}^{2} a_{4}^{2} J_{1} + a_{1}^{2} \left( {a_{2}^{2} J_{4} + a_{4}^{2} J_{2} \beta } \right)} \right]}}{{2a_{1} \left( {a_{2} + a_{4} } \right)\left( {a_{2} + a_{4} \beta } \right)}}$, unit is m^6^.

### Analysis of the out-plane force system and distortion differential equation of the box girder

By listing the force system balance equations on the left web, top and bottom plate, can obtain:

23 $$\left\{ \begin{gathered} q_{\rm{y}} = q_{{\rm{yA}}} + q_{{\rm{yD}}} = 2\frac{{a_{2} m_{{\rm{12}}} + a_{4} m_{{\rm{43}}} }}{{a_{2} a_{4} \sin \theta }} \hfill \\ q_{{\rm{x}1}} = q_{{\rm{xA}}} + q_{{\rm{xB}}} = 2\frac{{m_{{\rm{14}}} + m_{{\rm{41}}} }}{{a_{1} \sin \theta }} \hfill \\ q_{{\rm{x}2}} = q_{{\rm{xD}}} + q_{{\rm{xC}}} = 2\frac{{m_{{\rm{14}}} + m_{{\rm{41}}} }}{{a_{1} \sin \theta }} \hfill \\ \end{gathered} \right.$$

where *m*_12_, *m*_41_, *m*_43_, and *m*_14_ are the transverse bending moments at the end of each plate element. Equation (23) is substituted into $q_{{\text{y}}} + a_{{1}} q_{{{\text{x1}}}} /\left( {{2}a_{{4}} } \right) + a_{{1}} q_{{{\text{x2}}}} /\left( {{2}a_{{2}} } \right)$ of Eq. (22), and the relationship between the distortion angle *γ*_d_ and the transverse bending moment at the end of each plate is derived by using graph multiplication. The result can be written in the form shown in Eq. (24).

24 $$q_{\rm{y}} + \frac{{a_{1} q_{{\rm{x}1}} }}{{2a_{4} }} + \frac{{a_{1} q_{{\rm{x}2}} }}{{2a_{2} }} = \frac{{a_{1} (a_{2} + a_{4} )}}{{2a_{2} a_{4} \delta_{\rm{h}} }}[(a_{4} - a_{2} )X + 2h]\gamma_{\rm{d}}$$

Let $\frac{{a_{1} (a_{2} + a_{4} )}}{{2a_{2} \delta_{\rm{h}} }}[(a_{4} - a_{2} )X + 2h]\gamma_{\rm{d}}$ = *EI*_R_*γ*_d_, so $I_{\rm{R}} = \frac{{3a_{1} (a_{2} + a_{4} )[(a_{4} - a_{2} )X + 2h]}}{{a_{2} (K_{1} X^{2} - K_{2} X + K_{3} )}}$, unit is m^2^. where $K_{1} = \frac{{a_{4}^{3} }}{{4I_{4} }} + \frac{{a_{2}^{3} }}{{4I_{2} }} + \frac{{a_{1} (a_{4}^{2} + a_{2}^{2} + a_{2} a_{4} )}}{{2I_{1} }}$, $K_{2} = \frac{{a_{2}^{2} h}}{{I_{2} }} + \frac{{2a_{1} a_{2} h + a_{1} a_{4} h}}{{I_{1} }}$, $K_{3} = \frac{{a_{2} h^{2} }}{{I_{2} }} + \frac{{2a_{1} h^{2} }}{{I_{1} }}$.

Change the expression $\frac{{a_{1} P_{{\rm{d4}}} }}{{2a_{4} }} + \frac{{a_{1} P_{{\rm{d}2}} }}{{2a_{2} }} + P_{{\rm{d1}}}$ of Eq. (22) into $\frac{{a_{1} P_{{\rm{d4}}} }}{2} + \frac{{a_{1} a_{4} P_{{\rm{d}2}} }}{{2a_{2} }} + a_{4} P_{{\rm{d1}}}$. Substitute the distortion load obtained above, and make *Ω* = *a*_1_(*a*_2_ + *a*_4_)*/*(2h); then, it can be written as *PΩ*.

Through the above analysis, Eq. (22) can finally be written in the form of Eq. (25), which is based on the distortion angle *γ*_d_ of corner 4. The fourth-order distortion governing differential equation of the box girder with *γ*_d_ as an unknown quantity was established.

25 $$EI_{{\rm{\omega d}}} \gamma _{\rm{d}}^{{\prime \prime \prime \prime }} + EI_{\rm{R}} \gamma _{\rm{d}} = P\Omega$$

If the box girder is rectangular, then the distortion warping normal stress at point 1 of the box girder can also be written as $\sigma_{{\rm{d1}}} = \frac{{M_{\rm{c}} \beta h}}{{J_{1} (1 + \beta )}}$, can also be written as $\sigma_{{\rm{d1}}} = B_{{\text{d}}} \omega_{{\rm{d1}}} /I_{{\omega d}}$, where $\omega_{{\rm{d1}}} { = }\frac{{a_{4} h\beta }}{4(1 + \beta )}$, so $\omega_{{\rm{d4}}} { = }\frac{{a_{4} h}}{4(1 + \beta )}$. This is the box-girder distortion analysis Method 3.

## Solutions of distortion-controlled differential equations

The obtained distortion controlled differential equation can be solved by the initial parameter method as follows: let the initial parameter solution of the odd form of the differential equation be the following:

26 $$\gamma_{\rm{d}} = A_{1} \Gamma_{1} + A_{2} \Gamma_{2} + A_{3} \Gamma_{3} + A_{4} \Gamma_{4}$$

where *A*_*i*_ (*i* = 1, 2, 3, 4) is any integral constant, $\Gamma_{1} { = }\rm{sh(}\lambda z)\sin \rm{(}\lambda z)$, $\Gamma_{2} { = }\rm{sh(}\lambda z)\cos \rm{(}\lambda z)$, $\Gamma_{3} { = }\rm{ch(}\lambda z)\sin \rm{(}\lambda z)$, and $\Gamma_{4} { = }\rm{ch(}\lambda z)\cos \rm{(}\lambda z)$. When solving differential equations, the *I*_ωd_ and *I*_R_ of each method expression are different. The boundary conditions of the box girder are as follows: The fixed end constraint is $\gamma_{{\text{d}}} = \gamma_{{\text{d}}}^{\prime } = 0$; the constraint of the simply supported end with the rigid diaphragm is $\gamma_{{\text{d}}} = \gamma_{{\text{d}}}^{\prime \prime } = 0$; and the constraint of the free end with the rigid diaphragm is $\gamma_{{\text{d}}} = \gamma_{{\text{d}}}^{\prime \prime \prime } = 0$.

The distortion governing differential equation can also be solved by using the analogous beam on elastic foundation solutions (BEF solutions). The BEF method is a special form of the initial parameter solution, and the prerequisite for its use is *λL* > 2π, which is not restricted by the initial parameter solutions. When a unit distortion load is applied in the middle of the span of the box girder, the distortion angle of the box girder and the influence line of the distortion bimoment can be obtained. The BEF method solutions for Eq. (26) are shown in Eq. (27) as follows:

27 $$\left\{ \begin{gathered} \gamma_{\rm{d}} (z) = \frac{{\lambda e^{ - \lambda z} }}{{2EI_{\rm{R}} }}[\cos (\lambda z) + \sin (\lambda z)] \hfill \\ B_{\rm{d}} (z) = \frac{{e^{ - \lambda z} }}{4\lambda }[\cos (\lambda z) - \sin (\lambda z)] \hfill \\ \end{gathered} \right.$$

If *λL* ≤ 2π, which is solved by Eq. (28), then *z* is the longitudinal coordinate of each section of the box girder. The distortion angle of the simply supported box girder is *γ*_d_. The initial parameter expression of the distortion bimoment *B*_d_ is shown in Eq. (28), where *γ*_d0_ and *B*_d0_ are the distortion angle and distortion bimoment at *z* = 0 of the box girder, respectively, and their values can be determined by the corresponding boundary conditions.

28 $$\left\{ \begin{gathered} \gamma_{\rm{d}} (z) = \gamma_{{\rm{d}0}} \Gamma_{4} - \frac{{B_{{\rm{d0}}} \Gamma_{1} }}{{2\lambda^{2} EI_{{\omega d}} }} - \frac{{M_{{\rm{d0}}} (\Gamma_{3} - \Gamma_{2} )}}{{4\lambda^{3} EI_{{\omega d}} }} \, \hfill \\ B_{\rm{d}} (z){ = }\gamma_{{\rm{d0}}} 2EI_{{\omega d}} \lambda^{2} \Gamma_{1} + B_{{\rm{d}0}} \Gamma_{4} + \frac{{M_{{\rm{d0}}} (\Gamma_{2} + \Gamma_{3} )}}{2\lambda } \, \hfill \\ \end{gathered} \right.$$

## Numerical examples

**Figure 6:**
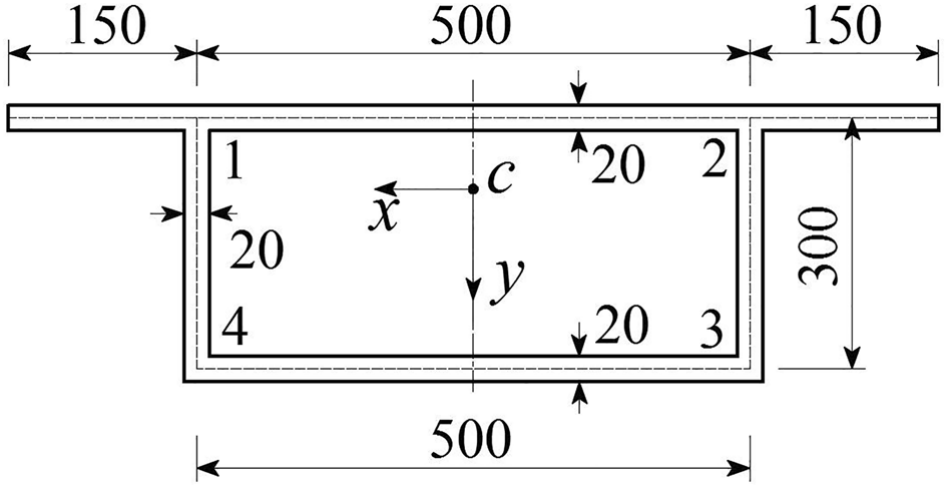
Cross section of the box girder (unit: cm).

Example 1, taken from the literature^2^, a simply supported box girder bridge with rectangular section, and a calculated span *L* = 80 m. *E* = 35 GPa, and *μ* = 0. A moment of 1 kNm is applied to the span of the top plate, and the cross-sectional dimensions are shown in Fig. 6. The calculated distortion characteristics and corner distortion warping normal stress are listed in Table 1. The values of *B*_d_ and *γ*_d_ in the table are calculated by the BEF solutions under a unit load, *σ*_d_ is the numerical value under the actual load, and its values are taken from the midspan section of the box girder.

**Table 1:** Distortional normal stress and characteristics of the box girder of example 1.

Items	*I*_ωd_/m^6^	*I*_R_/m^2^	*ω*_d4_/m^2^	*β*	*γ*_d_/(× 10^−10^ rad)	*B*_d_/(N m^2^)	*σ*_d1_/(KPa)	*σ*_d4_/(KPa)
Literature value^2^ ①	6.0850	0.002	2.54255	0.4749	0.6800	2.6280	0.5215	1.0981
Method 1 value ②	4.2712	0.007	2.54255	0.4749	0.0291	1.7579	0.4970	1.0464
Method 2 value ③	3.0432	0.001	2.54255	0.4749	0.6792	1.3140	0.5214	1.0978
Method 3 value ④	12.1728	0.004	2.54255	0.4749	3.3961	2.6279	0.5213	1.0978

In Table 1, the distortion characteristics of box girders obtained from the literature^2^ and various analysis methods are listed. The ratio of the distortion warping normal stress *β* and distortion sector coordinates *ω*_d_ values are equal, but there is a significant difference in the values of the transverse bending moment of inertia* I*_R_ and distortion warping sector moment of inertia *I*_ωd_ of the frame. This is caused by the different distortion analysis methods.

From Table 2, the maximum error between the distortion normal stress values of Method 1 and those of literature^2^ is 4.71%, while the distortion normal stress values of Method 2 and Method 3 and those of literature^2^ have very small errors. Using the results in literature^2^ as a reference, the advantages of the optimized methods in calculating the distortion effect of box girders can be verified. Compared to Method 1, Methods 2 and 3 have a simpler distortion analysis process and better calculation accuracy, but Method 1 can describe the concepts of box-girder distortion sector coordinates more comprehensively. Regardless of the analysis method, the numerical error in calculating the distortion normal stress of the box girder is very small.

**Table 2:** Distortional normal stress error.

Distortion normal stress	Error 1/% [(① − ②)/① × 100]	Error 2/% [(① − ③)/① × 100]	Error 3/% [(① − ④)/① × 100]
*σ*_d1_ (KPa)	4.70	0.02	0.04
*σ*_d4_ (KPa)	4.71	0.03	0.03

Example 2 is also taken from literature^2^, i.e., a simply supported box girder bridge with a trapezoidal cross section. The calculated span is *L* = 40 m, *E* = 34.3 GPa, the concentrated force acting in the span of the box girder is *P*_T_ = 1000 kN, and the eccentricity is *e* = 0.55 m. The calculation results of Methods 1 and 2 are listed in Table 3. Table 3 shows that the maximum error (*σ*_d_) of the numerical value does not exceed 5.39%. For the trapezoidal section box girder, Methods 1 and 2 are used to calculate the distortion effect of the box girder, and the *ω*_d_ values are not equal to each other, which is different from the rectangular box girder. In the analysis of example 1, although the *I*_ωd_ and *I*_R_ values are different, the *ω*_d_ values are the same. This shows the difference between rectangular and trapezoidal box girders in the calculation of distortion geometric characteristics.

**Table 3:** Distortional normal stress and characteristics of the box girder of example 2.

Items	*I*_ωd_/m^6^	*I*_R_/m^2^	*ω*_d4_/m^2^	*ω*_d1_/m^2^	*β*	*σ*_d1_/(MPa)	*σ*_d4_/(MPa)
Method 1 value	3.18040	0.02924	2.10617	0.82242	0.23429	0.04386	0.18722
Method 2 value	0.89353	0.00151	1.47989	0.34672	0.23429	0.04150	0.17713
Error/%	–	–	–	–	–	5.39	5.39

Example 3, i.e., a box girder with a trapezoidal cross section, is taken from literature^7^. The value of *σ*_d_ is generated by the actual load, and the calculated value is listed in Table 4. From the values in the literature^7^, the distortion values calculated by Methods 1 and 2 are not equal, but the calculated values of *σ*_d_ are basically the same, and the error is very small. Therefore, it can be considered that different methods are effective at calculating *σ*_d_ and that the values are equal, which is confirmed. In example 3, the *ω*_d_ values are not equal to each other.

**Table 4:** Distortional normal stress and characteristics of the box girder of example 3.

Items	*I*_ωd_/m^6^	*ω*_d4_/m^2^	*β*	*σ*_d1_/(MPa)	*σ*_d4_/(MPa)
Literature value^7^	0.02321	0.72000	–	–	23.35
Method 1 value	0.01611	0.65357	0.27742	6.195	22.33
Method 2 value	0.00574	0.50637	0.27742	6.294	22.69

**Figure 7:**
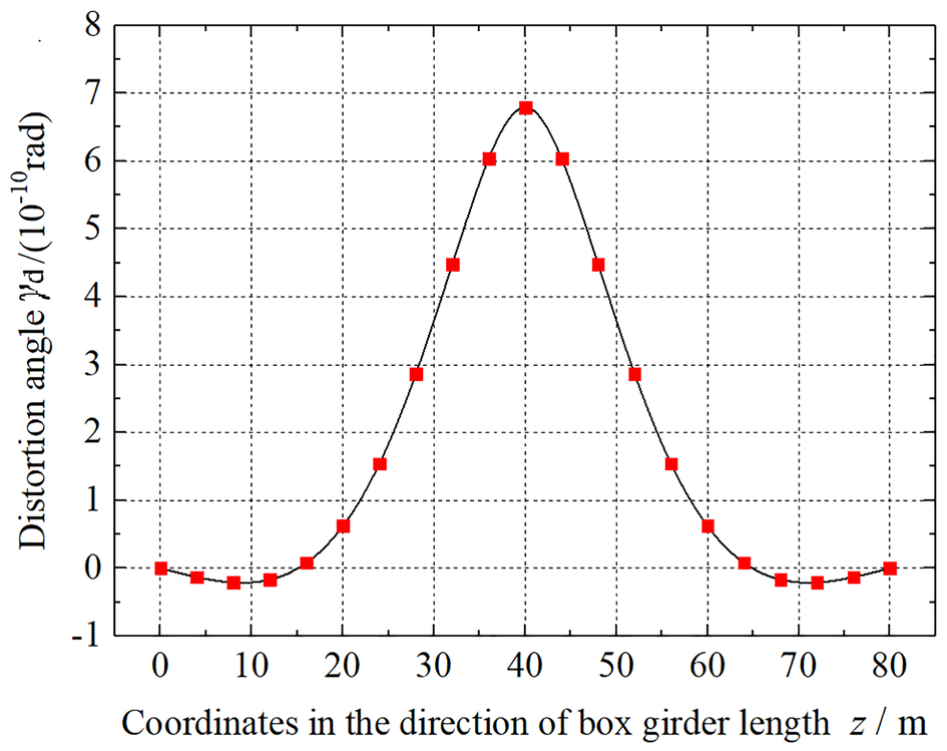
The variation in the distortion angle *γ*_d_ along the beam length.

**Figure 8:**
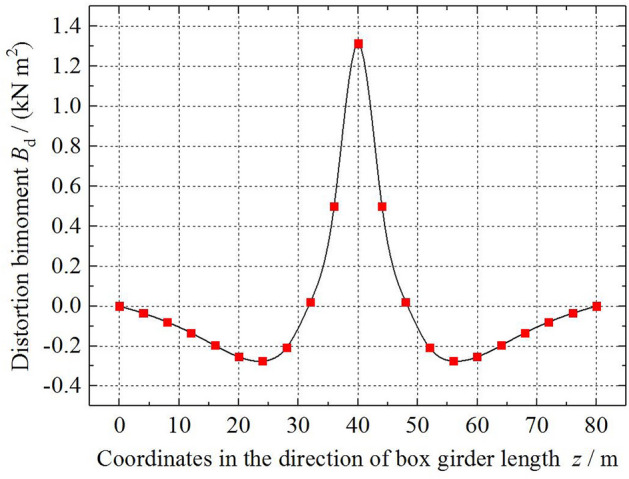
The variation in the distortion bimoment *B*_d_ along the beam length.

**Figure 9:**
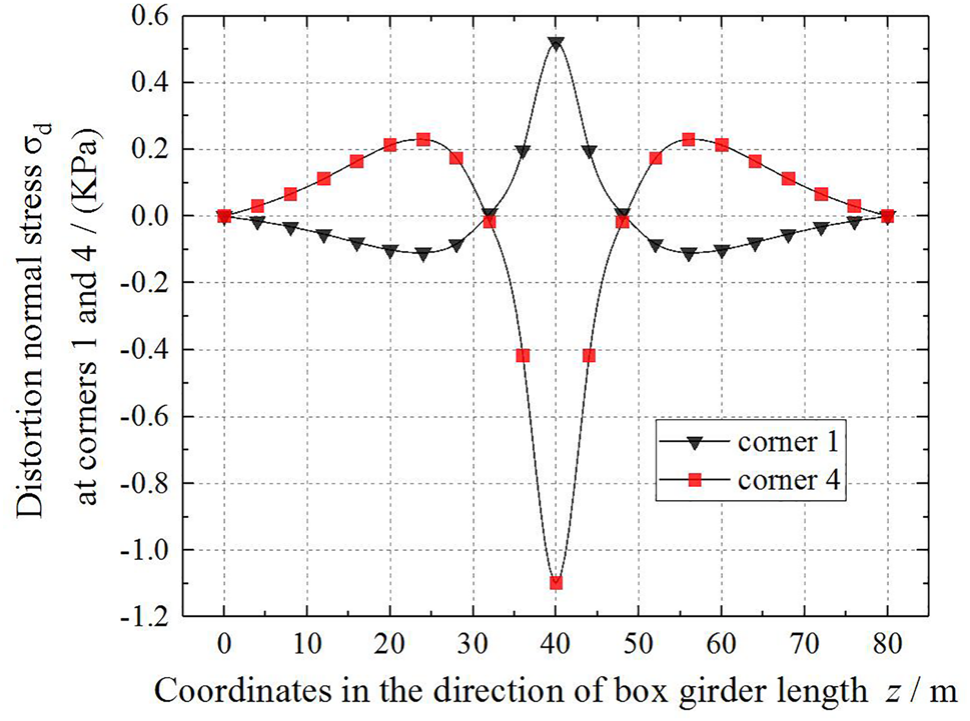
The variation in the distortion warping normal stress *σ*_d_ along the beam length.

**Figure 10:**
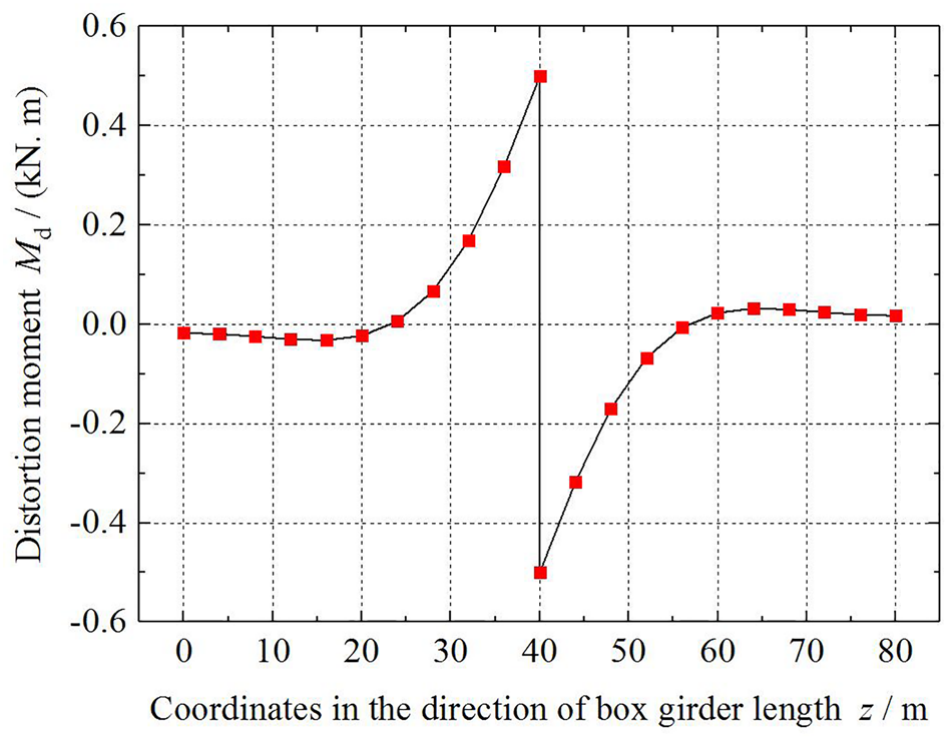
The variation in the distortion moment *M*_d_ along the beam length.

To study the distortion effect of the thin-walled box girder, Method 2 is adopted, and based on example 1, the calculation results under a unit concentrated load are obtained. The distribution and variation of *γ*_d_, *B*_d_, *σ*_d_ and *M*_d_ along the beam length are shown in Figs. 7, 8, 9 and 10. As shown in Fig. 7, the distortion angle *γ*_d_ has the maximum value at the mid-span section of the box girder, then gradually decreases, and exhibits the minimum value near *L*/8 or 7*L*/8 of the bridge span section. At the beam end, the distortion angle *γ*_d_ is 0. The value of distortion angle *γ*_d_ reflects the degree of change in the relative position of each plate of the box girder after distortion. For *γ*_d_ at the mid-span section of the box girder, the symbol is opposite to that near the *L*/8 or 7*L*/8 bridge span section, indicating that the deformation direction of the box girder section is opposite to that at the mid-span section in the area near the beam end.

Figure 8 shows the variation in the distortion bimoment *B*_d_ of the box girder along the length. At the mid-span section of the box girder, the distortion bimoment exhibits the maximum value, and then the value decreases rapidly. The length of this area is approximately *L*/8 of the box girder length. Different from the variation in the distortion angle along the box girder length, the distortion bimoment has a minimum value near the *L*/4–1.5*L*/4 (2.5*L*/4–3*L*/4) bridge span section. Compared with the variation in the distortion angle, the variation in the distortion bimoment *B*_d_ along the box girder length is much more dramatic, and the value changes from a positive maximum to a negative minimum in a small range of lengths.

The variation in the distortion warping normal stress *σ*_d_ of corners 1 and 4 of the box girder along the beam length is shown in Fig. 9. The variation law is similar to the distortion bimoment. In the area of a small mid-span section, the numerical symbol of *σ*_d_ is changed, because the diaphragm is set at the end of the box girder, and the distortion warping normal stress value gradually becomes 0. Figure 9 shows that the absolute value of the distortion warping normal stress peak at corner 1 is less than that at corner 4. Distortion warping has a greater impact on the bottom plate of the box girder than on the top plate. This is caused by a redistribution of distortion warping normal stress on the section due to the existence of the cantilever plate of the box girder. Figure 10 shows the distribution and variation of the distortion moment *M*_d_ of the box girder along the girder length. The absolute value of the distortion moment *M*_d_ is the largest at the mid-span section and then gradually decreases. It is not 0 at the end section of the box girder. The whole figure is antisymmetric about the mid-span section of the box girder.

## Conclusions

Through the optimization study of the existing box girder distortion analysis methods, several numerical examples are used to verify and compare the distortion geometric characteristics and other eigenvalues calculated by each method, and the distribution law of distortion effects such as distortion angle along the beam length is analysed. The conclusions are drawn as follows:

1. The analysis methods of box girder distortion research focus on different points, the analysis process is different, and the definition of the distortion angle is different, but the form of the distortion control differential equation is the same. The calculation of the distortion warping normal stress of the box girder by each method is basically the same, and the error is very small. For the three calculation examples in this paper, the maximum error is 5.39%. Regardless of the method, the corner distortion normal stress ratio *β* values are equal.
2. Through the optimization study of three distortion analysis methods, verification and comparison of numerical examples. Compared with method 1, the distortion theoretical analysis process of method 2 and method 3 is simpler, easy to be popularized in engineering practice, and meets the needs of engineering designers. Method 1 can describe the physical quantities such as distortion fan coordinates of box girder more completely, and the theoretical analysis process is more complete.
3. Due to the different theoretical concerns and analysis process, via three analysis methods, the calculation result of the distortion sector coordinates *ω*_d_ is affected by the cross-sectional form of the box girder. For a box girder with a rectangular cross section, the results via the three methods are the same, whereas for a box girder with the trapezoidal cross section, the results are different. The distortion warping sector moment of inertia *I*_ωd_ and transverse bending moment of inertia *I*_R_ of the frame are related to the numerical formation and the process of the various distortion analysis methods. *I*_ωd_, *I*_R_, and *ω*_d_, et al., show that the distorted geometric characteristics of the box girder are not the inherent characteristics of the box girder section, and the calculated values are not unique.
4. The distortion angle *γ*_d_ reflects the spatial relative position change in each plate of the box girder after distortion. For a simply supported box girder with only end diaphragms, under the action of a centralized distortion load, the longitudinal variation law of the distortion angle along the beam is inconsistent, and there is reverse deformation near the beam end. The distribution of the distortion warping normal stress along the beam length is the same as that of the distortion bimoment. Due to the existence of the box girder cantilever plate, the distribution of the distortion warping normal stress peak on the top plate is smaller than that on the bottom plate. The distribution of the distortion moment is antisymmetric with respect to the mid-span section of the box girder, and the value at the beam end is not 0.

## Data Availability

All data generated or analysed during this study are included in this published article.

## References

[1] Guo JQ, Fang ZZ, Zheng Z (2008). Design Theory of Box Bridges.

[2] Xiang HF, Yao LS (2001). Advanced Bridge Structure Theory.

[3] Huang JY, Xie X (2001). Structural Theory and Calculation Method of Urban Viaduct.

[4] Kermani B, Waldron P (1993). Analysis of continuous box girder bridges including the effects of distortion. Comput. Struct..

[5] Boswell LF, Li Q (1995). Consideration of the relationships between torsion, distortion and warping of thin-walled beams. Thin-Walled Struct..

[6] Jönsson J (1999). Distortional theory of thin-walled beams. Thin-Walled Struct..

[7] Zhang YH, Liu ZX, Lin LX, Zhou MD (2016). Analysis on distortion effect of thin-walled box girders based on principle of stationary potential energy. J. Cent. South Univ. (Sci. Technol.).

[8] Yoo CH, Kang J, Kim K (2015). Stresses due to distortion on horizontally curved tub-girders. Eng. Struct..

[9] Koleková Y, Kováč M, Baláž I (2017). Influence lines of bridges with box-girder cross-section under torsion and distortion. Procedia Eng..

[10] Marcello A, Michele FG (2016). Unified theory for analysis of curved thin-walled girders with open and closed cross section through HSA method. Eng. Struct..

[11] Dikaros IC, Sapountzakis EJ (2017). Distortional analysis of beams of arbitrary cross section using BEM. J. Eng. Mech..

[12] Sapountzakis EJ, Dikaros IC (2019). Advanced 3-D beam element including warping and distortional effects for the analysis of spatial framed structures. Eng. Struct..

[13] Shao JY, Zhang YH, Zhao QY, Yao XD (2019). Stress analysis on distortion of corrugated steel web box girders. Appl. Math. Mech..

[14] Zhang YH (2021). Analysis on distortion effect of box girders with inner-span diaphragms. China Civ. Eng. J..

[15] Wang ZN, Zhang YH (2022). Distortion effect of single box double-cell box girders with rectangular cross section. J. Southwest Jiaotong Univ..

[16] Li LF, Zhou C, Wang LH (2018). Distortion analysis of non-prismatic composite box girders with corrugated steel webs. J. Constr. Steel Res..

[17] Wright RN, Abdel-samad SR, Robinson AR (1968). BEF analogy for analysis of boxgirders. J. Struct. Div..

[18] Hsu YT, Fu CC, Schelling DR (1995). EBEF Method for distortional analysis of steel box girder bridges. J. Struct. Eng..

[19] Ren YZ, Cheng WM, Wang YQ, Chen QR, Wang B (2017). Distortional analysis of simply supported box girders with inner diaphragms considering shear deformation of diaphragms using initial parameter method. Eng. Struct..

[20] Wang ZN (2020). Research on Analytical Theory and Application of Transverse Internal Force of Box Girder.

